# Classical and next generation sequencing approaches unravel *Bymovirus* diversity in barley crops in France

**DOI:** 10.1371/journal.pone.0188495

**Published:** 2017-11-28

**Authors:** Mathieu Rolland, Julie Villemot, Armelle Marais, Sébastien Theil, Chantal Faure, Valérie Cadot, Romain Valade, Cindy Vitry, Frank Rabenstein, Thierry Candresse

**Affiliations:** 1 Groupe d'Etude et de contrôle des Variétés Et des Semences, Beaucouzé, France; 2 Unité Mixte de Recherche 1332 Biologie du Fruit et Pathologie, Institut National de la Recherche Agronomique, Université de Bordeaux, Villenave d’Ornon, France; 3 ARVALIS–Institut du végétal, Thiverval-Grignon, France; 4 Julius Kühn-Institut, Quedlinburg, Germany; GERMANY

## Abstract

Despite the generalized use of cultivars carrying the *rym4* resistance gene, the impact of viral mosaic diseases on winter barleys increased in recent years in France. This change could reflect i) an increased prevalence of the *rym4* resistance-breaking pathotype of *Barley yellow mosaic virus* Y (BaYMV-2), ii) the emergence of *rym4* resistance-breaking pathotypes of *Barley mild mosaic virus* (BaMMV) or iii) the emergence of other viruses. A study was undertaken to determine the distribution and diversity of viruses causing yellow mosaic disease. A collection of 241 symptomatic leaf samples from susceptible, *rym4* and *rym5* varieties was gathered from 117 sites. The viruses present in all samples were identified by specific RT-PCR assays and, for selected samples, by double-stranded RNA next-generation sequencing (NGS). The results show that BaYMV-2 is responsible for the symptoms observed in varieties carrying the resistance gene *rym4*. In susceptible varieties, both BaYMV-1 and BaYMV-2 were detected, together with BaMMV. Phylogenetic analyses indicate that the *rym4* resistance-breaking ability independently evolved in multiple genetic backgrounds. Parallel analyses revealed a similar scenario of multiple independent emergence events in BaMMV for *rym5* resistance-breaking, likely involving multiple amino acid positions in the viral-linked genome protein. NGS analyses and classical techniques provided highly convergent results, highlighting and validating the power of NGS approaches for diagnostics and viral population characterization.

## Introduction

The barley yellow mosaic disease has unexpectedly increased in prevalence in France over the past few years and is now an important concern in winter barley crops. Despite the extensive use of *rym4* resistant varieties, which afforded efficient protection during the past decade, yield losses are increasing and may reach up to 20% in some locations. In Europe and Asia, two potyviruses belonging to the genus *Bymovirus*, *Barley yellow mosaic virus* (BaYMV) and *Barley mild mosaic virus* (BaMMV), are responsible for this disease. These two soil-borne viruses have the typical bipartite genome of members of this genus [1, 2] and are transmitted by *Polymyxa graminis* [3, 4]. The genomic RNAs, RNA-1 and RNA-2, have respective sizes of around 7.5 and 3.5 kb and are translated into polyproteins which are cleaved and processed into functional proteins. BaYMV and BaMMV can remain active in the soil in the resting spores of *P*. *graminis*, and cannot therefore be controlled by pesticides. The only control strategy presently available is the use of resistant barley varieties [5]. Recessive resistance conferred by *rym* genes has been deployed against these two viruses. In Europe, most winter barley varieties carry the *rym4* gene and, more recently, a few varieties carrying the *rym5* gene have been deployed [6]. As for many other recessive resistance genes against *Potyviridae* members [7], the *rym4/rym5* alleles encode mutated versions of the host translation initiation factor eIF4E which are unable to support the virus cycle [8, 9].

The first record of barley yellow mosaic disease was reported in Japan [10] and the responsible virus was named BaYMV. In Asia, the disease was also found in China and South Korea [11, 12]. In Europe, the disease was first detected in Germany in 1977 [13] and later in other countries: France [14], the UK [15], Belgium [16], the Netherlands [17], Ukraine [18], Hungary [19], Italy [20], Greece [21], Spain [22], Bulgaria [23] and Poland [24]. The disease spread rapidly in Germany [25] and in the UK [26]. It was found that similar disease symptoms could be caused by two distinct viruses, named BaMMV and BaYMV [27]. The two viruses coexist in Europe [28–30] and BaMMV has also been detected in Japan [31]. Distinctions between BaMMV and BaYMV pathotypes were established according to their ability to infect barley cultivars carrying different resistance genes. In Japan, four BaYMV strains (BaYMV-I, II, III and IV) and two BaMMV strains (BaMMV-Ka1 and BaMMV-Na1) have been described [1, 32]. In Europe, the diversity of these viruses seems to be lower, with only two BaYMV (BaYMV-1 and BaYMV-2) and three BaMMV (BaMMV, BaMMV-Sil and BaMMV-Teik) strains identified so far [30, 33–35, 42].

In Europe, the use of barley varieties with resistance to the barley yellow mosaic complex began around 1989. The recessive resistance gene *rym4* from the landrace Ragusa was introduced in malting winter barley [36] and conferred resistance to the BaYMV-1 pathotype and to BaMMV [37]. For years, most winter barley cultivars in Europe have been protected by this resistance [9, 36]. A pathotype of BaYMV able to overcome the *rym4* resistance gene (BaYMV-2) was first detected in Germany and the UK, and then in other European countries [28, 34, 38, 39]. Limited at first to a few areas, this pathotype became later prevalent in some countries, such as in Germany, sometimes resulting in significant yield losses. The ability of BaYMV-2 to infect *rym4*-carrying varieties has been linked to a single amino acid change in the viral-linked genome protein (VPg) [40], a situation that parallels most other cases of resistance-breaking for eIF4E-based resistances [7].

A few barley varieties carrying the *rym5* resistance gene have been developed in an effort to limit the spread of BaYMV-2. However, isolates of BaMMV were rapidly reported in France [33], and in Germany [1, 41, 42] from *rym5* varieties, indicating that the ability to overcome this newly deployed resistance rapidly emerged within BaMMV populations. There is also a report of a BaYMV isolate found infecting *rym5* varieties in France [33]. The BaMMV-Sil and BaMMV-Teik genomes have been sequenced and explored in an effort to identify possible *rym5*-breaking mutations and again it was suggested, although in a less clear-cut fashion, that amino acid changes in the VPg region could be involved [35, 42]. The role of mutations in the VPg in *rym5*-breaking isolates was recently demonstrated in the case of Asian isolates of BaYMV, in which several simultaneous amino acid changes in the VPg were required for the virus to overcome the *rym5* resistance [43].

In addition to *rym 4/5* resistance-breaking virus isolates, other viruses can be responsible for soil-borne diseases with symptoms similar to those of the barley yellow mosaic disease. This is the case, of a new furovirus detected in the north-east of France (Marne) and for which the name Soil-borne wheat mosaic virus-Marne (SBWMV-Mar) was proposed [44]. According to its partial RNA-1 and RNA-2 sequences, this virus was found to be most closely related to Japanese isolates of SBWMV (SBWMV-JP), which were later considered as a tentative new species, distinct from SBWMV and for which the name *Japanese soil-borne wheat mosaic virus* (JSBWMV) has been proposed [45]. A similar virus has also been found in Germany and partially sequenced [46].

Faced with reports of increased prevalence of barley yellow mosaic disease and of increasing yield losses, the French barley producers and malting/brewing sector were in need of precise information to better address this rising challenge and to rethink future resistance genes deployment. The present study was therefore initiated with the objective to identify the viruses involved in the current surge of barley yellow mosaic disease, to clarify their distribution in France and to precise their ability to overcome the deployed *rym* resistance genes. A second objective was to explore the power of next-generation sequencing-based approaches to tackle such a problem and to compare the NGS-based results with those from more classical approaches.

## Materials and methods

### Plant material

**Fig 1:**
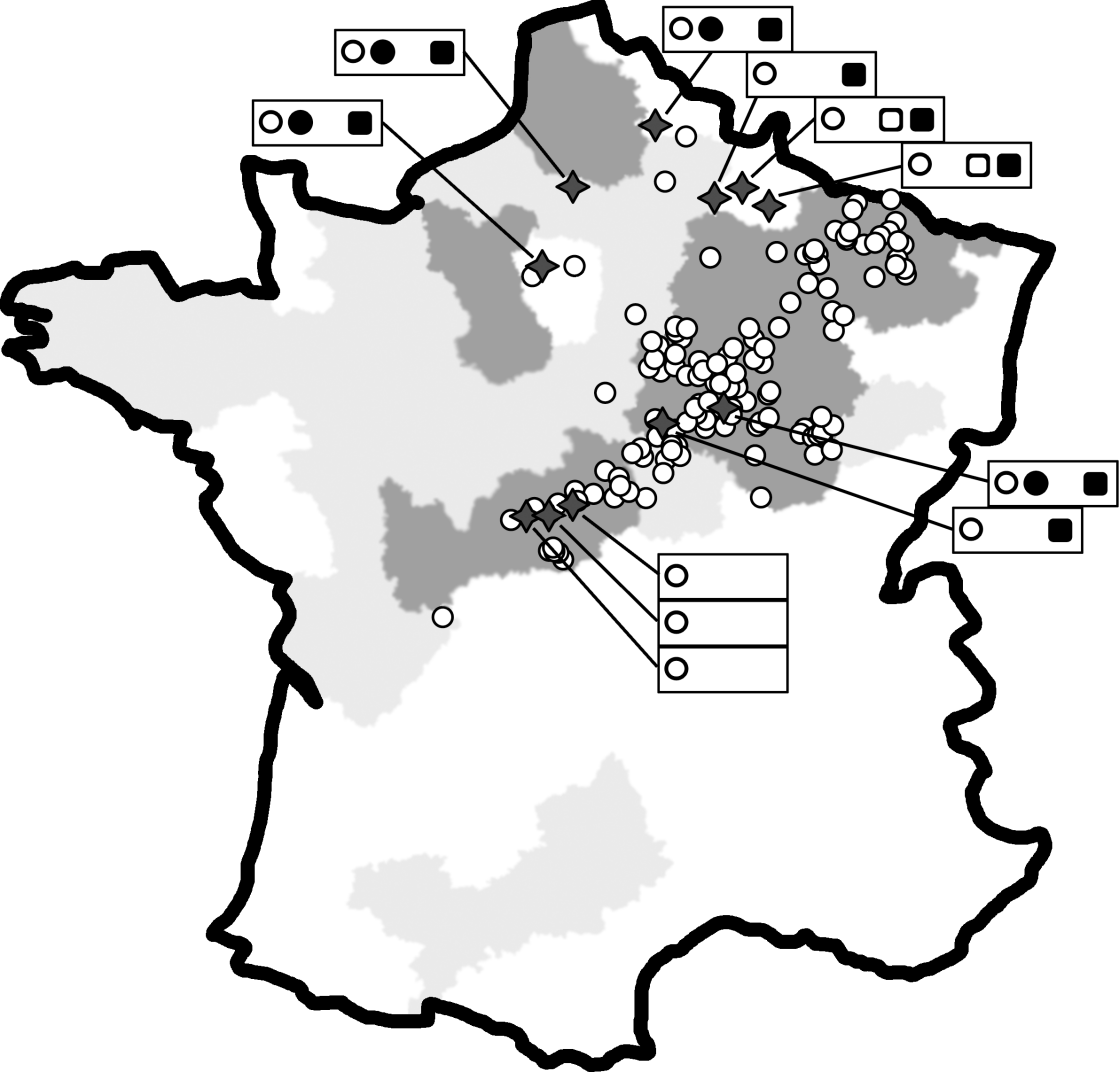
Main winter barley production areas in France, sampling sites used in the present study. Barley production areas are colour-coded according to barley acreage in each department: below 12500ha, white; from 12500 to 25000ha, pale grey; above 25000ha, dark grey (data: Agreste 2014). Each collection site of the survey is represented, and the differential-host experimental sites are shown as black stars. The *Bymovirus* diversity from these 11 sites is indicated as follows: BaYMV-1 as a black dot, BaYMV-2 as a white dot, BaMMV as a black square and *rym5*-breaking BaMMV as a white square.

Sampling was done at tillering stage (January to April) from 2013 to 2016, according to two different protocols, i) a survey of the virus(es) responsible for mosaic symptoms, ii) a differential host description of viral populations at selected infested sites. For the survey, leaves of *rym4-*carrying winter barley varieties presenting mosaic symptoms were collected from infested fields in the different French malting barley production areas (center, north and east France). A total of 151 samples (53 in 2013, 48 in 2014, 30 in 2015 and 20 in 2016) were collected from 106 sites (Fig 1). In the differential host description, reference susceptible (Plaisant and Orelie), *rym4-*carrying (Arturio, Esterel and Etincel) and *rym5-*carrying (Mosaic, Malice and a varieties under registration coded Var1, 2 and 3) barley varieties were grown in 11 infested sites (Fig 1). A total of 90 symptomatic samples (21 in 2013, 23 in 2014, 43 in 2015 and 3 in 2016) were collected: 48 and 31 from susceptible and *rym4* varieties respectively; 11 samples from *rym5* varieties were collected from two sites (Vouziers and Sorbon) in which symptoms were observed. Each sample was constituted of 20 leaves collected from ten plants (two leaves per plant). Samples were dried at room temperature and kept stored over anhydrous CaCl_2_ until further use, preserving the integrity of viral nucleic acids (single- or double-RNAs, DNA) in infected samples.

To extend the range of sequences available in the NGS study and in the BaMMV Vpg sequence analysis, five German samples from two sites (Bornum and Sunstedt) were included, one from a susceptible variety (Plaisant), one from a variety carrying *rym4* (Esterel) and three from varieties carrying *rym5* (Otto, Mosaic and Malice). These samples were collected, dried and stored using the described protocol.

### Molecular screening of samples for BaYMV/BaMMV and other barley viruses

For each sample, around 2cm of each collected leaves were ground into powder. Total RNA and DNA were extracted from 20mg of leaf powder, using the RNeasy Plant Mini kit (Qiagen, Hilden, Germany) and NucleoSpin Plant II kit (Macherey Nagel, Düren, Germany) respectively and following the manufacturer’s instructions. Reverse transcription was performed from 1μl of purified template RNAs using the Reverse Transcriptase Core kit (Eurogentec, Liège, Belgium) in a 10μl total volume, following the manufacturer’s instructions. PCR detection and identification of known bymoviruses and of other barley viruses were performed using the various protocols listed in Table 1. BaYMV-1 and BaYMV-2 typing was achieved either by Sanger sequencing (Genoscreen, Lille, France) of uncloned BaYMV VPg PCR products [47] or by using a dCAPS typing assay [48] once it became available (Table 1).

**Table 1:** Molecular methods used for the detection and identification of bymoviruses and other barley viruses.

Targeted viruses	PCR method	Primer sequence forward (F) and reverse (R) 5’ -> 3’ and annealing temperature (Tm) or reference
BaMMV and BaYMV	real-time PCR	Mumford et al. [49]
WDV *(a)* and BSMV *(b)*	PCR	Tao et al. [50]
B/CYDVs (PAV, MAV, RPV) *(c)*	PCR	Deb et al. [51]
BaMMV	PCR	Achon et al. [22]
HvEV *(d)*	PCR	• F : TGGATGAGGCTAACAGGCCA • R : GTCCATCGGTTTGTGGGCAA • Tm : 55°C
BaYMV-1 and BaYMV-2	dCAPS	Villemot & Rolland [48]
BaYMV	PCR + Sanger sequencing	Vaïanopoulos et al. [47]
BaMMV	PCR + Sanger sequencing	• F : TCTGGGTTTCTCCCAGGAAACA • R : CCATCTTTGCGCTGTCAATGGT • Tm : 53°C
BaYMV	PCR + Sanger sequencing	• F: AGCAAACTATGTGGCTTCAC • R: AAATTGGTCTTGAAGGCAA • Tm : 55°C

(a) Wheat dwarf virus

(b) Barley stripe mosaic virus

(c) Barley/Cereal yellow dwarf viruses

(d) Hordeum vulgare endornavirus

From *rym5* and susceptible varieties, the complete VPg coding region of BaMMV was amplified using the primers and the annealing temperature described in Table 1. Direct sequencing of the uncloned PCR products was performed by Genoscreen (Lille, France). The corresponding BaMMV VPg sequences have been deposited in Genbank with accession numbers KX117164 to KX117182 (S1 Table). Additionally, some samples were screened for the presence of barley endornavirus (HvEV) [52] using the primer set and the annealing temperature described in Table 1.

### Viral indexing through dsRNA next-generation sequencing

A complete viral indexing was performed on selected symptomatic barley samples through high throughput sequencing of cDNAs prepared from highly purified double stranded RNAs (dsRNAs) [53]. Samples were selected on the basis of the symptomatology and of the barley variety (and resistance gene content). In the early attempts it was found that HvEV, when present, represented a high proportion of viral reads detected and therefore reduced the coverage of the genome of other viruses present. Samples infected with HvEV, as determined using the PCR assay described above, were therefore excluded in later analyses. Samples were analyzed in multiplex format on 3 different runs of Illumina MiSeq pair-end sequencing (2x250nt), sometimes together with additional unrelated samples. Each MySeq run integrated between 12 and 22 samples (S2 Table). Following demultiplexing and quality trimming/filtering of reads, bioinformatic analysis of the sequencing data was performed essentially as described previously [53]. Information on the number of reads obtained for each sample is provided in S2 Table. Bioinformatic analyses involved either *de novo* assembly and annotation by BlastN and BlastX against Genbank, or mapping of reads against reference viral genomes using CLC Genomics workbench 7 or 8. Assembled BaMMV and BaYMV sequences showing more than 75% completeness were used for phylogenetic analyses and have been deposited in Genbank (Accession numbers KX117183 to KX117209 and KX831456 to KX831469, S1 Table).

### Viral sequences comparisons and phylogenetic analyses

Multiple sequence alignments of contigs obtained from NGS data and of BaMMV and BaYMV sequences (or of deduced encoded proteins) and of European and Asian isolates retrieved from Genbank were performed using the ClustalW algorithm [54] as implemented in MEGA 6.0 [55]. Phylogenetic trees were reconstructed in MEGA 6.0, using strict nucleotide or amino acid distances and either the Neighbour Joining (NJ) or the Maximum Likelihood (ML) algorithms. Branch support was evaluated by bootstrap analysis (500 replicates).

## Results

### Barley mosaic disease in France on *rym4* varieties is caused by BaYMV-2

From 2013 to 2016 a survey was conducted in the main French malting barley production areas. In 106 sites, 151 leaf samples were collected from *rym4* carrying varieties showing mosaic symptoms. Each sample was tested by RT-PCR assays for the presence of BaYMV and BaMMV. Pathotyping of BaYMV isolates was performed by Sanger sequencing or dCAPS. 101 of the 151 collected samples were also tested by PCR for other frequent barley-infecting viruses: WDV, BSMV, BYDV-MAV, BYDV-PAV and CYDV-RPV. The results obtained are provided in Table 2.

**Table 2:** Number of samples tested and found positive to bymoviruses and other barley-infecting viruses. All viruses were detected by specific PCR or RT-PCR assays. BaYMV typing was performed using PCR product sequencing or a dCAPS typing assay [48].

Study	rym	Bymoviruses				BaYMV type				Other barley viruses			
		Sample number	BaYMV	BaYMV+ BaMMV	BaMMV	Sample number	BaYMV-1	BaYMV-1+2	BaYMV-2	Sample number	WDV	BSMV	BYDV
Survey	rym4	151	145	0	0	145	0	0	145	101	0	0	4
Differential hosts study	rym4	31	31	0	0	31	0	0	31	16	0	0	0
	-	48	23	24	1	47	2	6	39	25	0	0	3
	rym5	11	0	0	11	na	na	na	na	3	0	0	0

na: does not apply.

WDV and BSMV were not detected in any of the analyzed samples, while BYDV was detected in only four samples among the 101 tested (three samples containing BYDV-MAV, two samples containing BYDV-PAV in coinfection with BYDV-MAV, one sample containing CYDV-RPV). This low prevalence of C/BYDV likely reflects the selection of samples with yellow mosaic disease symptoms rather than the true prevalence of C/BYDV in winter barley crops. BaMMV was not detected in any of the symptomatic *rym4* samples tested, in accordance with the resistance conferred against this virus by the *rym4* gene. BaYMV-2 was detected in 145 out of 151 tested samples (Table 2).

In order to obtain a more precise description of the presence of the various barley-infecting bymoviruses in France, a set of reference differential barley varieties composed of susceptible and of *rym4-* and *rym5*-resistant varieties were planted in 11 locations (Fig 1) and 90 symptomatic samples were collected from 2013 to 2016. Each sample was tested by RT-PCR assays for the presence of BaYMV and BaMMV. 44 of the 90 collected samples (16 from *rym4*, three from *rym5* and 25 from susceptible varieties) were also tested by PCR for WDV, BSMV, BYDV-MAV, BYDV-PAV and CYDV-RPV. The results obtained are provided in Table 2.

Neither WDV nor BSMV were detected in any of the samples, but BYDV was detected in three samples (12%) among the 25 tested from the susceptible varieties Plaisant and Orelie. BYDV-MAV was found in all three samples, in mixed infection with BYDV-PAV in two of them. Testing of the symptomatic samples of *rym4* varieties provided the same observations as the survey: no BaMMV was detected and BaYMV-2 was found in all 31 analyzed samples (Table 2). The findings were different for samples from the susceptible varieties Plaisant and Orelie, which revealed the presence of both bymoviruses. BaYMV was observed in 98% of the samples (two BaYMV-1 (4.2%), 39 BaYMV-2 (83%) and six BaYMV-1/2 mixed infections (12.8%)) and BaMMV in 52% of them (50% in mixed infection with BaYMV and 2% in single infection; Table 2). Overall, BaYMV-1 was found in four out of the 11 study sites, while BaMMV was found in all of them, demonstrating that even if they are not detected in *rym4* varieties, BaYMV-1 and BaMMV are still present in French barley production areas.

### Broad viral indexing of selected barley samples using dsRNA next-generation sequencing

In order to rule out the possibility that the observed symptoms might be caused by other viral agent(s) than those detected by the various specific PCR assays used, selected barley samples corresponding to susceptible varieties and to resistant varieties carrying the *rym4* or *rym5* resistance genes (Table 3) were analyzed by next generation sequencing using highly purified dsRNAs as the target [53]. Highly variable number of reads were obtained for the various samples (S2 Table), but given the very high enrichment in viral sequences afforded by dsRNAs purification, analysis of the sequencing data allowed a reliable identification of the viruses present, including for the phloem-limited BYDV. Besides BaYMV and BaMMV, the only detected plant viruses were BYDV (in two samples, in coinfection with BaYMV and/or BaMMV) and HvEV [52, 56] in six samples. Although not providing a definite proof, this result largely rules out the possible involvement of other RNA viruses in the observed symptoms.

**Table 3:** Indexing of selected symptomatic barley samples by next generation sequencing. NGS analyses were performed on highly purified double-stranded RNAs extracted from 21 barley leaf samples. The viruses identified are indicated, with the percentages of total reads mapping to their genome; PCR and NGS results are compared for BaYMV and BaMMV screening. BaYMV type, either Y1 (BaYMV-1) or Y2 (BaYMV-2), was determined from codon at position 132 of VPg in NGS data and in parallel with the dCAPS assay.

Sample code	Variety	*rym*	BaYMV		BaMMV		BYDV	JSBWMV	HvEv	BaYMV NGS typing	BaYMV dCAPS typing
			% of reads	PCR	% of reads	PCR	% of reads	% of reads	% of reads		
MO-13-67C	Plaisant	-	20.2%	+	39.3%	+	0%	0%	0.1%	Y1: AAG (K)	Y1 + Y2
MO-14-022C	Plaisant	-	24.1%	+	0%	-	0%	0%	0.2%	Y2: AAC (N) + CAT/C (H)	Y2
MO-14-022S	Plaisant	-	38.8%	+	3.5%	+	0%	0%	0%	Y1: AAG (K)	Y1
MO-14-032C	Plaisant	-	65.0%	+	0%	-	0%	0%	0%	Y2: AAC (N)	Y2
MO-14-124C	Plaisant	-	70.0%	+	4.0%	+	0%	0%	0%	Y1: AAG (K)	Y1
MO-15-109C	Plaisant	-	77.0%	+	0.1%	-	2.1%	0%	0%	Y2: AAC (N)	Y2
MO-15-133C	Plaisant	-	20.0%	+	41.7%	+	0.5%	0%	0.1%	Y2: AAC (N)	Y2
MO-15-160C	Plaisant	-	21.1%	+	4.6%	+	0%	0%	0%	Y2: AAC (N)	Y2
MO-15-221C	Plaisant	-	82.3%	+	0%	-	0%	0%	0%	Y2: AAT (N) + CAG (Q)	Y2
MO-15-257C	Plaisant	-	52.3%	+	31.9%	+	0%	0%	0%	Y1: AAG (K) + Y2: AAC/T	Y1
MO-15-280C *	Plaisant	-	27.6%	+	47.1%	+	0%	0%	0%	Y2: AAC/T (N) + Y1: AAG (K)	Y2
MO-15-367C	Plaisant	-	31.3%	+	38.4%	+	0%	0%	0%	Y2: AAC (N)	Y2
MO-13-6C	Arturio	*rym4*	93.9%	+	0%	-	0%	0%	0%	Y2: AAC (N)	Y2
MO-13-10C	Etincel	*rym4*	26.0%	+	0%	-	0%	0%	68.2%	Y2: AAC (N)	Y2
MO-13-26C	Arturio	*rym4*	90.6%	+	0%	-	0%	0%	1.0%	Y2: CAT (H)	Y2
MO-13-74C	Etincel	*rym4*	10.7%	+	0%	-	0%	0%	73.7%	Y2: AAC (N) + CAC (H)	Y2
MO-15-217C	Esterel	*rym4*	81.7%	+	0%	-	0%	0%	0%	Y2: AAC (N)	Y2
MO-15-407C *	Esterel	*rym4*	69.8%	+	0%	-	0%	6,2%	0%	Y2: AAT/C (N)	Y2
MO-15-140C	Mosaic	*rym5*	0%	-	82.7%	+	0%	0%	0%	na	na
MO-15-186C	Var3	*rym5*	0%	-	49.3%	+	0%	0%	0%	na	na
MO-15-415C *	Otto	*rym5*	0.1%	-	32.8%	+	0%	18.6%	0%	na	na

*: Samples from Germany; na: not applicable.

In this table, the cumulated percentage of viral reads is less than 100% because of the presence of remaining cellular and other un-mapped reads.

There was an excellent correlation between the results of NGS-based indexing and those from the virus-specific PCR assays. For BaMMV and BaYMV, only two discrepancies were observed. In both cases, a virus not detected by RT-PCR was detected as about 0.1% of NGS reads (on the order of 35 to 100-fold less than for any other BaYMV or BaMMV-infected sample (Table 3).

As expected, no BaMMV was detected in the samples carrying the *rym4* gene. In these samples, the detected BaYMV isolates coded for an asparagine or for a histidine at position 132 of the VPg (Table 3), amino acids that have been associated with the BaYMV-2 pathotype and its ability to overcome the *rym4* resistance [40].

In contrast, samples originating from susceptible varieties that do not carry any of the *rym* genes showed more variability at that position, with either an AAG codon encoding a lysine (BaYMV-1, non resistance-breaking), a range of codons encoding an asparagine or a histidine (BaYMV-2, resistance-breaking) or, in three samples, mixes of the BaYMV-1 and BaYMV-2 codons (Table 3). This result suggests that although BaYMV-2 is prevalent, BaYMV-1 isolates are still present in a range of environments in France.

**Fig 2:**
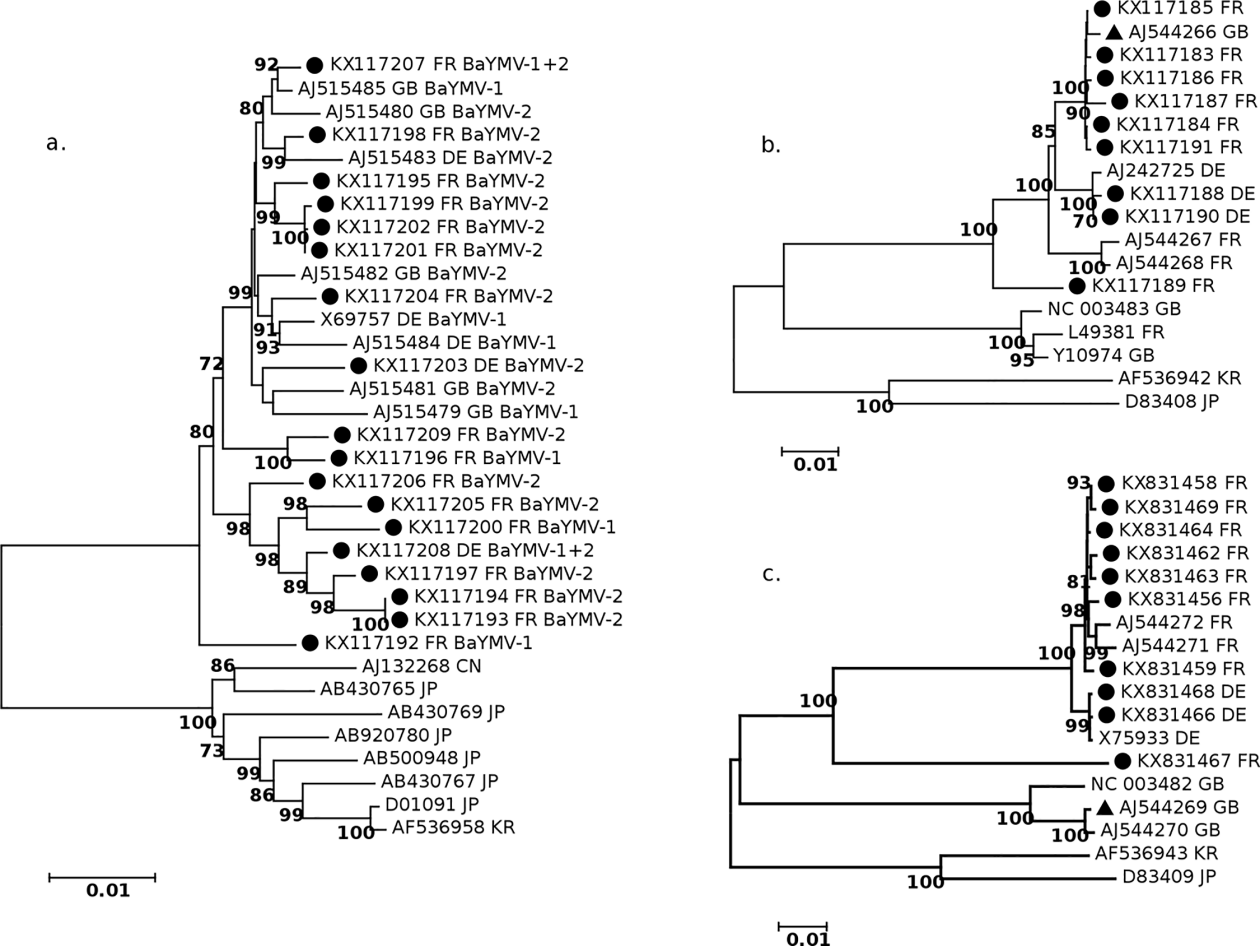
**Phylogenetic trees reconstructed for BaYMV RNA-1 (a.), BaMMV RNA-1 (b.) and BaMMV RNA-2 (c.).** Neighbour joining trees were built from complete RNA-1 and RNA-2 sequences from this study (black dots) and from additional sequences retrieved from Genbank. Bootstrap values >70% are shown. The country of origin of the isolate is indicated with a two letter code (CN = China, DE = Germany, FR = France, GB = Great Britain, JP = Japan, KR = Korea). Accession numbers are given and, for European isolates, the BaYMV type, either BaYMV-1 or BaYMV-2 is shown. The sequences of the UK isolate which appears to be pseudorecombinant are marked by a black triangle.

Complete or near complete genomic sequences for both genomic RNAs of BaYMV and/or BaMMV could be reconstructed from the NGS data of most analyzed samples and were used to compare the isolates found in France with those analyzed in the past or present in other countries and for which genomic sequences are available in Genbank. Phylogenetic trees were reconstructed using either Neighbor Joining (NJ) or Maximum Likelihood strategies and showed essentially similar topologies, so that only NJ trees are shown. In the case of BaYMV, two major clusters of isolates can be discriminated, one corresponding to European isolates and the other to Asian isolates (Fig 2A). Despite the existence of statistically supported subclusters within the European cluster, no particular grouping of isolates according either to their country of origin or to their resistance-breaking status could be detected in the RNA1 tree (Fig 2A) and a similar observation could be made from the RNA2 tree (results not shown). The situation with BaMMV is different; three major clusters are observed for both RNAs, one from Asia and the two others from Europe (Fig 2B and 2C); furthermore, a geographic subclustering of isolates, separating the German isolates from the ones from France and the UK can be detected (Fig 2B and 2C). In addition, comparison of the phylogenetic trees reconstructed for the RNA1 and for the RNA2 provides evidence for pseudorecombination between the two European clusters (Fig 2B and 2C): indeed, the UK-L isolate [35] clusters with English isolates in the RNA2 tree (AJ544269; Fig 2C) while clustering with the French-German isolates in the RNA1 tree (AJ544266; Fig 2B).

### Identification and characterization of BaMMV isolates able to overcome the *rym5* resistance

In addition to susceptible and *rym4* varieties, Barley varieties carrying the *rym5* resistance gene were also grown as differential hosts in the 11 selected sites. As expected, in most locations, these *rym5* varieties showed no symptoms. However, mosaic symptoms were observed on these varieties in two sites, in Vouziers and Sorbon, in the north east of France. Only BaYMV-2 and BaMMV had been detected in susceptible varieties at these two sites and all *rym5* samples tested positive for BaMMV using the virus-specific PCR assays. The ability of some BaMMV isolates to multiply and accumulate in *rym5* varieties was also confirmed by the dsRNA NGS analysis (Table 3).

**Fig 3:**
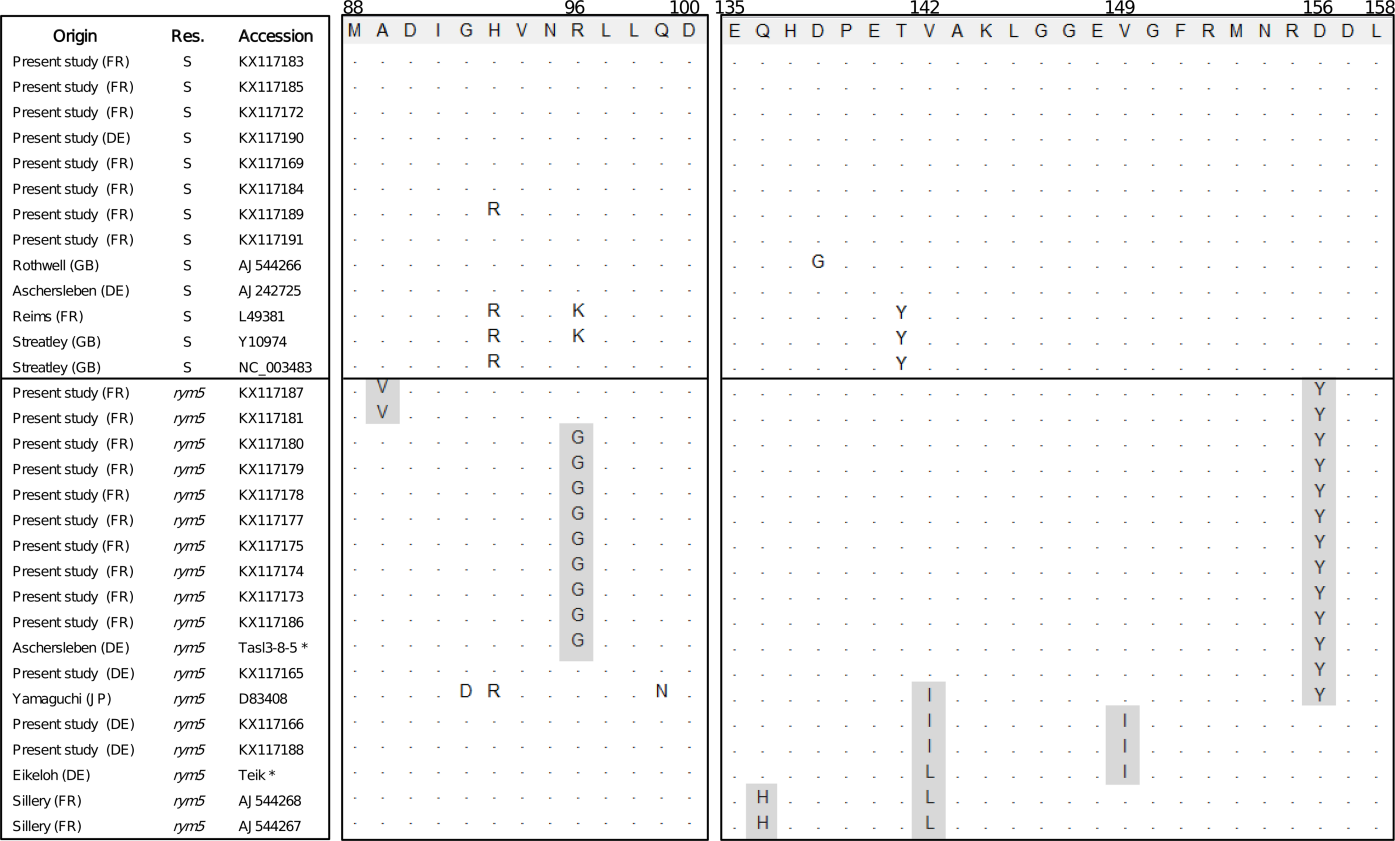
VPg amino acid sequences of BaMMV isolates found in susceptible varieties and in *rym5* carrying varieties. The Location column indicates the country of origin using a two-letter code: DE = Germany, FR = France, GB = Great Britain, JP = Japan. The resistance column (Res.) indicates whether the variety of origin is susceptible (S) or carries *rym5* resistance (*rym5*). Accession numbers are provided. Amino acids identical to the reference sequence highlighted at the top are indicated by dots. Mutations specific to *rym5*-breaking isolates and shared by at least two isolates are shaded. * The Tasl3-8-5 and Teik isolate sequences are provided in [42].

Further analyses were undertaken with *rym5-*breaking BaMMV isolates. Considering that *rym5* encodes a modified version of eIF4E [8, 9], and that the viral VPg has been shown to be involved in breaking this type of resistance [7, 43], the complete sequence of the VPg gene of ten BaMMV isolates detected on symptomatic *rym5* varieties was determined. For comparison purposes, the VPg gene sequence of eight BaMMV isolates detected from susceptible varieties were also determined. Four additional samples from Germany were also studied to extend the range of sequences available. The corresponding amino acid sequences were aligned and compared, together with the sequences of reference isolates available in Genbank. No single mutation specific for the *rym5*-breaking isolates could be identified (Fig 3), suggesting that several amino acid positions might independently or in combination contribute to the overcoming of *rym5* by BaMMV isolates.

## Discussion

Using two sampling protocols from 2013 to 2016, classical detection and characterization techniques (RT-PCR, Sanger sequencing, dCAPS) and NGS-based approaches, this study provides a description of the viruses responsible for mosaic symptoms on winter barley in the main French malting barley production areas.

Using the survey collection of 151 symptomatic leaf samples, of symptomatic barley varieties carrying resistance gene *rym4*, from 106 sites, PCR analyses revealed the absence of BaMMV and a very high prevalence (96%) of BaYMV. The six symptomatic samples in which BaYMV was not detected likely showed abiotic stress symptoms that may have been mistaken with yellow mosaic symptoms as no other virus was detected. NGS analyses in selected symptomatic samples confirmed the presence of BaYMV isolates harboring resistance-breaking mutations in their VPg and excluded the presence of other RNA viruses. The ability of the dsRNA-based NGS approach to detect other viruses, if present, is demonstrated by the detection of BYDV in one sample and of JSBWMV in two samples originating from Germany (Table 3). Taken together, these results demonstrate that in France, the resistance gene *rym4* has retained its effectiveness against BaMMV viral populations but that *rym4*-breaking BaYMV-2 isolates are now widely distributed and responsible for the current re-emergence of barley mosaic disease. This is similar to what was reported in Germany or Belgium where BaYMV-2 is now predominant [57].

The dominant presence of BaYMV-2 in leaves of *rym4* carrying varieties does not necessarily reflect the viral diversity found in the soil, as *P*. *graminis* acts like a long term reservoir for soil-borne viruses [58]. The susceptible reference varieties Plaisant and Orelie were grown in 11 selected locations. PCR assays and NGS analyses of symptomatic samples from these varieties demonstrate the existence of a larger virus diversity than observed in *rym4* varieties. Interestingly, BaYMV-1, BaYMV-2 and BaMMV were detected, with BaYMV-2 being the most frequent (93.9% of samples, 11/11 test sites, Fig 1) followed by BaMMV (52%, 8/11 sites) and by BaYMV-1 (16.7%, 4/11 sites). These results highlight the extent to which BaYMV-1, which was the most prevalent viral form in the 1990’s and early 2000’s has been replaced by BaYMV-2. The very wide adoption of *rym4*-carrying varieties has undoubtedly contributed to this result. If they are to have any chances of success in the future, the ongoing efforts to develop new resistant varieties should take into account the whole viral diversity present in soils, and in particular the frequent presence of BaMMV.

The analysis of BaYMV RNA-1 and RNA-2 phylogenies failed to reveal any particular clustering of isolates of the large European group on the basis of either geographical origin or resistance breaking properties. Considering that multiple resistance-breaking mutations are observed at VPg position 132, sometimes in mixed populations, and that resistance-breaking isolates are observed throughout the large European cluster, BaYMV’s virulence must have evolved in many locations and from diverse BaYMV-1 genetic backgrounds. This multiple evolution scenario is also in accordance with the limited diffusion ability of the *P*. *graminis* vector.

As for BaYMV, in BaMMV RNA-1 and RNA-2 phylogenies, no clustering of isolates based on the resistance genes harbored by the isolation host is observed. However, a subclustering of German isolates is observed, as well as a clustering of isolates from the UK (Fig 2B and 2C). The UK isolates are very divergent from other European isolates with the exception of an isolate from Reims in France (L49381). But the observation that the UK-L isolate has a RNA-1 clustering with isolates from continental Europe suggests the possibility of exchanges and of pseudorecombination between these clusters. Since BaYMV and BaMMV share essentially the same biology and transmission properties, it is surprising to observe that European populations of BaMMV are geographically structured while populations of BaYMV are not. This observation may reflect differences in the evolutionary history of these two closely related viruses or in their dispersal ability. Alternatively, it may also be a spurious observation, resulting from the selection of sequenced BaMMV isolates to date, which may dissipate as more sequences become available.

The *rym5* gene confers resistance to BaYMV-1, BaYMV-2 and BaMMV and has been considered as a potential alternative to *rym4* in case of *rym4* resistance breakdown. BaMMV isolates able to overcome *rym5* have been described in the past in several countries [32, 42, 59]. They have also been reported in France, more precisely in the Marne area in Sillery [33]. In the present study, BaMMV accumulation in symptomatic *rym5* varieties was observed by both specific PCR and NGS analysis in two of the 11 study sites, in Vouziers and Sorbon, which are not very far from Sillery. A similar observation of *rym5* breakdown was also made in samples from two sites in Germany (Bornum and Sunstedt). The VPg sequence of additional BaMMV isolates (*rym5*-breaking or not) was determined by Sanger sequencing or obtained from NGS data, in an effort to identify mutations that might be associated with resistance-breaking (Fig 3). The VPg was selected for this analysis because the VPg generally harbors the mutations allowing the overcoming of resistances mediated by eIF4E [7, 60, 61] and was recently demonstrated to be the protein accumulating resistance-breaking mutations in the case of Asian BaYMV *rym5*-breaking isolates [43].

Contrary to the situation with *rym4*-breaking BaYMV isolates, but in accordance with the recent analysis of *rym5*-breaking BaYMV isolates [43], no single mutation in the VPg appears to be specific to all *rym5*-breaking BaMMV isolates (Fig 3). Mutations at positions 96 (Lys or Arg to Gly), 142 (Val to Ile or to Leu) and 156 (Asp to Tyr) are frequently observed and specific of the *rym5-*breaking isolates (Fig 3). It is therefore quite possible that, alone or in combination with additional mutations at positions 89 (Ala to Val), 136 (Gln to His) and 149 (Val to Ile), these mutations could confer the ability to overcome the *rym5* resistance.

This hypothesis is supported by the observation that these mutations are shared by geographically distant and phylogenetically unrelated (Fig 2) isolates, which have probably evolved independently towards virulence. Taken together, the results make a rather strong case for the involvement of these mutations in *rym5-*breaking. However, we cannot rule out the possibility that other regions of the genome than the VPg could be involved, as was demonstrated in the case of the overcoming of the eIF4E-based *mo1* gene for resistance to *Lettuce mosaic virus*, which may involve the C-terminal portion of the CI helicase [62]. Only experiments performed with full-length BaMMV infectious clones may in time allow confirming or rejecting these various hypotheses.

The geographic structuration of BaMMV populations and the fact that *rym5*-breaking isolates have been observed in several countries and in isolates belonging to all phylogenetic clusters strongly suggest that, as for BaYMV evolution towards *rym4*-breaking, the *rym5*-breaking capacity has independently evolved in a range of BaMMV populations and can therefore emerge in a broad range of genetic backgrounds. This highlights the need for the analysis and use of more durable sources of resistance against BaYMV and BaMMV such as *rym1/rym11*, *rym18* or other genes that have been described.

In the context of development of NGS for diagnostic purposes, it is interesting to consider the input of this technique in the presented study. Results of the BaYMV and BaMMV specific PCR detection assays and the dsRNA-based NGS analyses were highly parallel. The two cases of discrepancies (no detection by RT-PCR with NGS detection representing about 0.1% of total reads: 35 to 100X less than in any other infected sample) might reflect viral concentrations below the limit of detection of the RT-PCR assays, a low level contamination in the NGS analysis or uneven viral distribution in the composite samples used. The strain-typing results were similarly highly convergent between the NGS and dCAPS analyses; with discrepancies corresponding to situations where mixed infections were missed by one or the other of the techniques. Taken together, these results provide a detailed comparison and validation of NGS based-approaches for the detection and characterization of bymoviruses in barley samples. In addition, the use of the NGS-based approach allowed to rule out the involvement of other viruses in the symptoms that prompted the study and generated BaYMV and BaMMV sequences that were instrumental in the elaboration of the multiple evolution scenario of resistance-breaking. Overall, by providing the sequences of the viral isolates involved and in excluding the presence of unknown RNA viruses, NGS technology improved considerably the description of causal viral populations.

## Supporting information

S1 Table Accession numbers of sequences deposited in Genbank. (DOCX)

S2 Table Information on MySeq multiplexed runs, sequencing reads and reads mapping to BaYMV VPg for the various samples analyzed in this study. (DOCX)
