# Supplementary material for: Classical and next generation sequencing approaches unravel Bymovirus diversity in barley crops in France
Source: PLoS One. 2017 Nov 28;12(11):e0188495. doi: 10.1371/journal.pone.0188495 (PMC5705140; doi:10.1371/journal.pone.0188495)
Supplement: S1 Table — (DOCX) [file pone.0188495.s001.docx]

| **Code** | **Variety** | ***rym*** | **Accession numbers** |
| --- | --- | --- | --- |
|  |  |  |  |
| MO-13-67C | Plaisant | - | KX117196 (BaYMV RNA-1); KX117184 (BaMMV RNA1) ; KX831458 (BaMMV RNA2) |
| MO-14-022C | Plaisant | - | KX117198 (BaYMV RNA1) |
| MO-14-022S | Plaisant | - | KX117183 (BaMMV RNA1); KX117192 (BaYMV RNA1) ; KX831456 (BaMMV RNA2) |
| MO-14-032C | Plaisant | - | KX117199 (BaYMV RNA1) |
| MO-14-124C | Plaisant | - | KX117200 (BaYMV RNA1) ; KX831459 (BaMMV RNA2) |
| MO-15-109C | Plaisant | - | KX117206 (BaYMV RNA1) |
| MO-15-133C | Plaisant | - | KX117185 (BaMMV RNA1); KX831462 (BaMMV RNA2) |
| MO-15-160C | Plaisant | - | KX117202 (BaYMV RNA1) |
| MO-15-221C | Plaisant | - | KX117205 (BaYMV RNA1) |
| MO-15-257C | Plaisant | - | KX117189 (BaMMV RNA1); KX117207 (BaYMV RNA1); KX831467 (BaMMV RNA2) |
| MO-15-280C | Plaisant | - | KX117190 (BaMMV RNA1); KX117208 (BaYMV RNA1); KX831468 (BaMMV RNA2) |
| MO-15-367C | Plaisant | - | KX117191 (BaMMV RNA1); KX831469 (BaMMV RNA2) |
| MO-15-160C | Plaisant | - | KX117172 (BaMMV VPg) |
| MO-14-124C | Plaisant | - | KX117169 (BaMMV VPg) |
| MO-13-6C | Arturio | *rym4* | KX117193 (BaYMV RNA1) |
| MO-13-10C | Etincel | *rym4* | KX117194 (BaYMV RNA1) |
| MO-13-26C | Arturio | *rym4* | KX117195 (BaYMV RNA1) |
| MO-13-74C | Etincel | *rym4* | KX117197 (BaYMV RNA1) |
| MO-15-217C | Esterel | *rym4* | KX117204 (BaYMV RNA1) |
| MO-15-407C | Esterel | *rym4* | KX117203 (BaYMV RNA1) |
| MO-15-140C | Mosaic | *rym5* | KX117186 (BaMMV RNA1); KX831463 (BaMMV RNA2) |
| MO-15-186C | *Var3* | *rym5* | KX117187 (BaMMV RNA1); KX831464 (BaMMV RNA2) |
| MO-15-415C | Otto | *rym5* | KX117188 (BaMMV RNA1); KX831466 (BaMMV RNA2) |
| MO-15-185C | *Var2* | *rym5* | KX117181 (BaMMV VPg) |
| MO-15-159C | *Var3* | *rym5* | KX117180 (BaMMV VPg) |
| MO-15-158C | *Var2* | *rym5* | KX117179 (BaMMV VPg) |
| MO-15-157C | *Var1* | *rym5* | KX117178 (BaMMV VPg) |
| MO-15-141C | Malice | *rym5* | KX117177 (BaMMV VPg) |
| MO-13-97C | Malice | *rym5* | KX117175 (BaMMV VPg) |
| MO-13-95C | *Var3* | *rym5* | KX117174 (BaMMV VPg) |
| MO-13-48C | Malice | *rym5* | KX117173 (BaMMV VPg) |
| MO-14-130C | Mosaic | *rym5* | KX117165 (BaMMV VPg) |
| MO-14-184C | Malice | *rym5* | KX117166 (BaMMV VPg) |
