# Supplementary material for: Classical and next generation sequencing approaches unravel Bymovirus diversity in barley crops in France
Source: PLoS One. 2017 Nov 28;12(11):e0188495. doi: 10.1371/journal.pone.0188495 (PMC5705140; doi:10.1371/journal.pone.0188495)
Supplement: S2 Table — (DOCX) [file pone.0188495.s002.docx]

| **Sample code** | **Variety** | ***rym*** | **Run number** | **Samples in run** | **MID** | **Primer sequence** | **# read** | **Reads mapped on BaYMV VPg** | **Average coverage BaYMV VPg** |
| --- | --- | --- | --- | --- | --- | --- | --- | --- | --- |
|  |  |  |  |  |  |  |  |  |  |
| MO-13-67C | Plaisant | - | 1 | 12 | MID-GENCO15 | **CAAGCAGG**TGTGTTGGGTGTGTTTGG | 589,705 | 144 | 40.8x |
| MO-14-022C | Plaisant | - | 1 | 12 | MID-GENCO33 | **GCTCTACC**TGTGTTGGGTGTGTTTGG | 1,277,822 | 2,202 | 696.2x |
| MO-14-022S | Plaisant | - | 1 | 12 | MID-GENCO1 | **AACCGCAA**TGTGTTGGGTGTGTTTGG | 794,759 | 1,341 | 411.4x |
| MO-14-032C | Plaisant | - | 1 | 12 | MID-GENCO34 | **GCTGCGGT**TGTGTTGGGTGTGTTTGG | 296,876 | 681 | 219.9x |
| MO-14-124C | Plaisant | - | 1 | 12 | MID-GENCO36 | **GGTACTCC**TGTGTTGGGTGTGTTTGG | 65,824 | 116 | 39.5x |
| MO-15-109C | Plaisant | - | 3 | 22 | MID-GENCO36 | **GGTACTCC**TGTGTTGGGTGTGTTTGG | 81,571 | 356 | 124.5x |
| MO-15-133C | Plaisant | - | 2 | 16 | MID-GENCO15 | **CAAGCAGG**TGTGTTGGGTGTGTTTGG | 39,338 | 97 | 30.3x |
| MO-15-160C | Plaisant | - | 2 | 16 | MID-GENCO34 | **GCTGCGGT**TGTGTTGGGTGTGTTTGG | 34,073 | 116 | 37.1x |
| MO-15-221C | Plaisant | - | 3 | 22 | MID-GENCO34 | **GCTGCGGT**TGTGTTGGGTGTGTTTGG | 35,559 | 284 | 102.7x |
| MO-15-257C | Plaisant | - | 3 | 22 | MID-GENCO38 | **GTCAACGG**TGTGTTGGGTGTGTTTGG | 294,983 | 551 | 201.7x |
| MO-15-280C * | Plaisant | - | 3 | 22 | MID-GENCO43 | **TCTATGAC**TGTGTTGGGTGTGTTTGG | 400,098 | 372 | 126.3x |
| MO-15-367C | Plaisant | - | 3 | 22 | MID-GENCO48 | **TTGCGTCA**TGTGTTGGGTGTGTTTGG | 63,210 | 97 | 33.2x |
| MO-13-6C | Arturio | *rym4* | 1 | 12 | MID-GENCO3 | **AACTAGTA**TGTGTTGGGTGTGTTTGG | 1,561,408 | 4,566 | 1745.3x |
| MO-13-10C | Etincel | *rym4* | 1 | 12 | MID-GENCO8 | **AGGCGCCT**TGTGTTGGGTGTGTTTGG | 547,892 | 105 | 31.1x |
| MO-13-26C | Arturio | *rym4* | 1 | 12 | MID-GENCO12 | **ATTAGCTA**TGTGTTGGGTGTGTTTGG | 331,929 | 1,960 | 728.5x |
| MO-13-74C | Etincel | *rym4* | 1 | 12 | MID-GENCO18 | **CCAACCAT**TGTGTTGGGTGTGTTTGG | 585,664 | 139 | 43.8x |
| MO-15-217C | Esterel | *rym4* | 3 | 22 | MID-GENCO18 | **CCAACCAT**TGTGTTGGGTGTGTTTGG | 99,000 | 373 | 135.1x |
| MO-15-407C * | Esterel | *rym4* | 3 | 22 | MID-GENCO3 | **AACTAGTA**TGTGTTGGGTGTGTTTGG | 260,935 | 1,119 | 396.5x |
| MO-15-140C | Mosaic | *rym5* | 2 | 16 | MID-GENCO21 | **CGATAGAG**TGTGTTGGGTGTGTTTGG | 49,617 | 0 | - |
| MO-15-186C | *Var3* | *rym5* | 2 | 16 | MID-GENCO36 | **GGTACTCC**TGTGTTGGGTGTGTTTGG | 15,240 | 0 | - |
| MO-15-415C * | Otto | *rym5* | 3 | 22 | MID-GENCO14 | **CAAGAGTT**TGTGTTGGGTGTGTTTGG | 132,400 | 3 | 1.2x |

*: Samples from Germany
